# The Roles of the Catalytic and Noncatalytic Activities of Rpd3L and Rpd3S in the Regulation of Gene Transcription in Yeast

**DOI:** 10.1371/journal.pone.0085088

**Published:** 2013-12-17

**Authors:** Daniella Yeheskely-Hayon, Anat Kotler, Michal Stark, Tamar Hashimshony, Shira Sagee, Yona Kassir

**Affiliations:** Department of Biology, Technion, Haifa, Israel; National Cancer Institute, United States of America

## Abstract

In budding yeasts, the histone deacetylase Rpd3 resides in two different complexes called Rpd3L (large) and Rpd3S (small) that exert opposing effects on the transcription of meiosis-specific genes. By introducing mutations that disrupt the integrity and function of either Rpd3L or Rpd3S, we show here that Rpd3 function is determined by its association with either of these complexes. Specifically, the catalytic activity of Rpd3S activates the transcription of the two major positive regulators of meiosis, *IME1* and *IME2*, under all growth conditions and activates the transcription of *NDT80* only during vegetative growth. In contrast, the effects of Rpd3L depends on nutrients; it represses or activates transcription in the presence or absence of a nitrogen source, respectively. Further, we show that transcriptional activation does not correlate with histone H4 deacetylation, suggesting an effect on a nonhistone protein. Comparison of *rpd3*-null and catalytic-site point mutants revealed an inhibitory activity that is independent of either the catalytic activity of Rpd3 or the integrity of Rpd3L and Rpd3S.

## Introduction

Histone deacetylation plays a pivotal role in the regulation of transcription. Deacetylation of lysine residues in the N-terminal tail domain of histones H3 and H4 correlates with transcriptional repression of most promoters, while transcriptional activation correlates with acetylation of these residues [1-4]. In some cases, histone deacetylation also correlates with transcriptional activation (for reviews and specific examples see 5,6). These activities were revealed using global chromatin immunoprecipitation (ChIP)-on-chip DNA microarray assays [7-9], as well as by direct gene- specific analysis. For example, in budding yeast, activation of osmoregulated genes depends on histone deacetylation through the recruitment of the histone deacetylase Rpd3 by the mitogen-activated protein kinase (MAPK) Hog1 [10]. Similarly, in mammals, deacetylation of cytokine-inducible genes such as interferon–β is essential for enhancing transcription [11].

All eukaryotic genomes contain genes encoding histone deacetylases (HDACs) that reside in large heterogeneous complexes [7,12-15]. In yeast, the HDAC Rpd3 is present in small (0.6 MDa) and large (1.2 MDA) complexes called Rpd3S and Rpd3L, respectively, that include unique and common subunits [7,14]. These complexes are recruited to specific sites on DNA as follows: Rpd3L is recruited to promoters in association with specific transcription factors such as Ume6, Ash1, Ime1, Whi5, and Stb1 [13,16-18]. At the promoter region, Rpd3 deacetylates specific lysine residues on histones H3 and H4 in a localized region spanning about two nucleosomes [19]. In contrast, Rpd3S is recruited to the open reading frame (ORF) by RNA polymerase II upon its phosphorylation by Cdk7/Kin28 on Ser5 within its carboxy-terminal domain [20]. Two of its specific components, Eaf3 and Rco1, recruits it to a methylated lysine residue of histone H3, a modification carried out by Set2 complexed with RNA polymerase II [21,22]., it recruits Rpd3S Nonetheless, Rpd3S is also recruited to the promoter of *HSP82* [23].The specific subunits of these complexes may affect the stability of the complex or perform different catalytic functions such as histone methylation or chromatin remodeling [21,22,24]. Sin3 and Ume1 are shared by each complex [21,22]. Sin3 functions as a scaffold to assemble different proteins, and it is required to target the complex to specific promoters by its association with specific DNA-binding proteins [15,25,26]. For example, in *S. cerevisiae*, Sin3 associates with the Zn-cluster protein Ume6 [19]. Further, HDAC function can lead to global repression via a nonspecific mechanism [27].

The molecular mechanism by which deacetylation activates transcription is still an enigma [5]. Transcriptional modulation may depend on dual roles of specific subunits present in the HDAC complexes [21,22,24]. For example, the Eaf3 subunit of the yeast Rpd3S complex is also a subunit of which is required for spreading of silenced chromatin, is facilitated by the acetylation of histone H4 lysine residue 12. Because this lysine residue is an Rpd3 substrate, it is possible that in cells deleted for *RPD3* the expected increase in acetylation leads to increased Sir3 binding and consequently reduced transcription. This is one example explaining how Rpd3 functions as a positive regulator [8].

In the budding yeast *Saccharomyces cerevisiae*, initiation of meiosis depends on nitrogen depletion in the absence of glucose and the presence of a nonfermentable carbon source such as acetate. Nitrogen depletion activates a transcriptional cascade, which is roughly divided into genes expressed at early (EMG), middle (MMG), and late (LMG) times during meiosis, which are all controlled by the master regulator Ime1. In *S. cerevisiae*, the histone deacetylase Rpd3 functions dynamically in the transcriptional repression and activation of *IME1*, EMGs, MMGs, and LMGs. For example, Rpd3 functions as a positive regulator of *IME1* transcription early in meiosis and as a negative regulator that is required to inhibit *IME1* transcription during late meiosis [16]. The effect is direct, because both Rpd3 and Sin3 are recruited by Ime1 to the IREu element in its promoter [16], to which Ime1 itself is recruited by the transcription factor Msn2/4 [28]. Rpd3 functions as a negative regulator of EMGs following recruitment to their promoters by Ume6 [19]. Ume6 binds to a specific sequence (URS1), which is present and active in all EMGs as well as in other genes not involved in meiosis [29-31]. Rpd3 functions as a positive, crucial regulator of the transcription of MMGs, LMGs, and the early-middle gene *NDT80*, which encodes the direct transcriptional activator of MMGs [32]. It is not known if Rpd3 binds to the promoters of these genes.

Our aim here was to determine how Rpd3 exhibits opposite functions when regulating genes encoding the components of different meiotic networks and how its affects on the transcription of a specific gene are modulated during different stages of meiosis. We postulated that the Rpd3L and Rpd3S might possess distinct functions. We show that Rpd3L and Rpd3S possess specific functions as follows: the catalytic activity of Rpd3 present in Rpd3S is required for the efficient transcription of *IME1* and *IME2* (representative EMGs) during vegetative growth with acetate as the sole carbon source (SA medium) or upon nitrogen depletion with acetate as the sole carbon source (SPM medium, meiotic conditions). This complex also activates the transcription of *NDT80* but only in SA medium. In contrast, in SA medium, Rpd3L functions as a negative regulator for all genes, whereas upon nitrogen depletion it functions as a positive regulator early during meiosis. Moreover, we reveal a novel function of Rpd3, which is independent of its presence in intact Rpd3L or Rpd3S that repressed the transcription of all meiosis-specific genes during late meiosis; and in SA medium, it repressed transcription of only *IME1* and *IME2*. Finally, we show that the essential positive role of Rpd3 on the transcription of *NDT80* is not mediated through an effect on the pachytene checkpoint, a noncoding antisense RNA, or on the function of either Ndt80 or Sum1, which activate and repress the transcription of *NDT80*, respectively.

## Materials and Methods

### Strains and Plasmids

The relevant genotypes of the isogenic strains and plasmids used in this study are listed in Tables 1 and 2, respectively. Details on how these strains and plasmids were constructed are available upon request.

**Table 1:** List of Strains.

	Relevant genotype	Remarks, Reference
	MATa haploids	
Y1064	*ura3-52*, *leu2,3-112*, *trp1*Δ, *his3*Δ*::hisG*, *ade2-1*, *metX*, *gal80*Δ*::hisG*, *gal4*Δ*::hisG*	[66]
Y1043	*ime1*Δ::*hisG*, *leu2,3-112::LEU2-ime1*(-3.2 to +200)-*lac*Z	Y1064 derivative using P1408 and YIp1875
Y1075	*ime1*Δ::*hisG*	Y1064 derivative using YIp1408
Y1179	*leu2,3-112::LEU2-GAL1uas-HIS4uas-his4-lacZ*	Y1064 derivative using YIp2218
Y1214	*leu2,3-112::LEU2-UASru-his4-lacZ*	Y1064 derivative using YIp2102
Y1332	*ume6*Δ::*hisG*	Y1064 derivative using P1583
Y1381	*leu2,3-112::LEU2-GAL1uas-HIS4uas-his4-lacZ*, ume6Δ::*hisG*	Y1332 derivative using YIp2218
Y1535	*rpd3*Δ::*HIS3*, *ime1*Δ::*hisG*,	Y1043 derivative using
	*leu2,3-112::LEU2-ime1(-3.2 to +200)-lacZ*	YIp2566
Y1762	*ndt80*Δ*C’*::*IME2p-NDT80-TRP1*, *ime1::hisG*	Y1075 derivative using YIp3078
Y1765	*rpd3*::*RPD3-13xmyc-URA3*	Y1064 derivative using YIp3081
Y1813	*rco1*Δ::URA3, *leu2,3-112::LEU2-GAL1uas-HIS4uas-his4-lacZ*	Y1179 derivative using p3109
Y1827	*sum1*Δ::*URA3*, *leu2,3-112::LEU2-UASru-his4-lacZ*	Y1214 derivative using P3153
Y1843	*sds3*Δ::*HIS3*, *leu2,3-112::LEU2-GAL1uas-HIS4uas-his4-lacZ*	Y1179 derivative using P3144
Y1846	*rpd3*Δ::*HIS3*, *rad17*Δ::*URA3*	Y1535 derivative using YIp3158
Y1879	*rpd3*Δ::*HIS3*	Y1064 derivative using YIp2566
Y1881	*rco1*Δ::*URA3*, *sds3*Δ::*HIS3*, *leu2,3-112::LEU2-GAL1uas-HIS4uas-his4-lacZ*	Y1813 derivative using P3144
Y1893	*dep1*Δ::*hisG*, *leu2,3-112::LEU2-GAL1uas-HIS4uas-his4-lacZ*	Y1179 derivative using P3215
Y1913	*sum1*Δ::*URA3*, *rpd3*Δ::*HIS3*, *leu2,3-112::LEU2-UASru-his4-lacZ*	Y1827 derivative using P2566
Y1943	*sds3*::*SDS3-13myc-tADH1-HIS3*	Y1064 derivative using YIp3182
Y1948	*sds3*::*SDS3-13myc-tADH1-HIS3*, *rco1*::*RCO1-6HA-k1TRP1*	Y1943 derivative using YIp3179
Y2057	*rpd3*::*HIS3*, *trp1*::*TRP1-RPD3*	Y1879 derivative using YIp3315
Y2060	*rpd3*Δ::HIS3, *trp1*::TRP1- *rpd3H150AH151*	Y1879 derivative using YIp3316
	*MAT*α haploids	
Y1065	*ura3-52*, *trp1*Δ, *leu2-3,112*, *his3*Δ*::hisG*, *ade2-R8*, *gal80*Δ*::hisG*, *gal4*Δ*::hisG*	[66]
Y1328	*ume6*Δ::*hisG-URA-hisG*	Y1065 derivative using P1583
Y1536	*rpd3*Δ::*HIS3*	Y1065 derivative using YIp2566
Y1761	*ndt80* Δ*C’*::*IME2p-NDT80-TRP1*	Y1065 derivative using YIp3078
Y1766	*rpd3*:: *RPD3-13xmyc-URA3*	Y1065 derivative using YIp3081
Y1816	*rco1*Δ::*URA3*	Y1065 derivative using P3109
Y1844	*sds3*Δ::*HIS3*	Y1065 derivative using P3144
Y1847	*rpd3*Δ::*HIS3*, *rad17*Δ::*URA3*	Y1536 derivative using YIp3158
Y1877	*sum1*Δ::*URA3*	Y1065 derivative using YIp3153
Y1878	*sum1*Δ::*URA3, rpd3*Δ::*HIS3*	Y1877 derivative using YIp2566
Y1880	*rpd3*Δ::*HIS3*	Y1065 derivative using YIp2566
Y1882	*rco1*Δ::*URA3*, *sds3*Δ::*HIS3*	Y1816 derivative using P3144
Y1892	*dep*1Δ::*hisG*	Y1065 derivative using P3215
Y1944	*sds3*::*SDS3-13myc-tADH1-HIS3*	Y1065 derivative using YIp3182
Y1949	*sds3*::*SDS3-13myc-tADH1-HIS3*, *rco1*::*RCO1-6HA-k1TRP1*	Y1944 derivative using YIp3179
Y2058	*rpd3*::*HIS3*, *trp1*::*TRP1-RPD3*	Y1880 derivative using YIp3315
Y2061	*rpd3*Δ::HIS3, *trp1*::TRP1- *rpd3H150AH151*	Y1880 derivative using YIp3316
	*MAT*a/*MAT*α diploids	
Y1631	wild type	Y1064 X Y1065
Y1388	*ume6*Δ::*hisG*/*ume6*Δ::*hisG*-*URA3-hisG*,	Y1381 x Y1328
	*leu2-3,112*/*leu2,3-112::LEU2-GAL1uas-HIS4uas-his4-lacZ*	
Y1537	*rpd3*Δ::*HIS3*/*rpd3*Δ::*HIS3*, *ime1*Δ::*hisG*/*IME1*, *leu2-3,112/ leu2-3,112::LEU2-ime1(-3.2 to +200*)*- lacZ*	Y1535 x Y1536
Y1763	*ndt80*Δ*C’*::*IME2p-NDT80-TRP1*/*ndt80* Δ*C’* :: *IME2p-NDT80-TRP1*, *ime1*Δ::*hisG*/*IME1*	Y1761 x Y1762
Y1767	*rpd3*::*RPD3-13xmyc-URA3*/*rpd3*::*RPD3-13xmyc-URA3*	Y1765 x Y1766
Y1814	*rco1*Δ::*URA3*/*rco1*Δ::*URA3*, *leu2,3-112*/*leu2,3-112::LEU2::GAL1uas-HIS4uas-his4-lacZ*	Y1813 x Y1816
Y1845	*sds3*Δ::*HIS3*/*sds3*Δ::*HIS3*, *leu2,3-112*/*leu2-3,112::LEU2-GAL1uas-HIS4uas-his4-lacZ*	Y1843 x Y1844
Y1848	*rpd3*Δ::*HIS3*/*rpd3*Δ::*HIS3*, *rad17*Δ::*URA3*/*rad17*Δ::*URA3*	Y1846 x Y1847
Y1870	*rpd3*Δ::*HIS3*/*rpd3*Δ::*HIS3*, *NDT80*/ *ndt80*Δ*C’*::*IME2p-NDT80-TRP1*	Y1537 derivative using YIp3078
Y1883	*rco1*Δ::*URA3*/*rco1*Δ::*URA3*, *sds3*Δ::*HIS3*/*sds3*Δ:: *HIS3*, *leu2,3-112/leu2-3,112::LEU2::GAL1uas-HIS4uas-his4-lacZ*	Y1881 x 1882
Y1884	*leu2,3-112/leu2-3,112::LEU2::GAL1uas-HIS4uas-his4-lacZ*	Y1065 x Y1179
Y1888	*rpd3*Δ::*HIS3*/*rpd3*Δ::*HIS*	Y1879 x Y1880
Y1894	*dep1*Δ::*hisG*/*dep1*Δ::*hisG*, *leu2,3-112/leu2-3,112::LEU2::GAL1uas-HIS4uas-his4-lacZ*	Y1892 x Y1893
Y1914	*sum1*Δ::*URA3*/*sum1*Δ::*URA3*, *rpd3*Δ::*HIS3*/*rpd3*Δ ::*HIS3*, *leu2,3-112::LEU2-UASru-his4-lacZ/leu2-3,112,*	Y1878 x Y1913
Y1950	*sds3*:: *SDS3-13myc-tADH1-HIS3/sds3*::*SDS3-13myc-tADH1-HIS3*, *rco1*::*RCO1-6HA-k1TRP1*/*rco1*::*RCO1-6HA-k1TRP1*	Y1948 x Y1949
Y2059	*rpd3*Δ::*HIS3*/*rpd3*Δ::*HIS3*, *trp1*::*TRP1*-*RPD3*/ *trp1*::*TRP1-RPD3*	Y2057 x Y2058
Y2062	*rpd3*Δ::HIS3/*rpd3*Δ::HIS3, *trp1*::TRP1- *rpd3H150AH151*/*trp1::TRP1-rpd3H150AH151*	Y2060 X Y2061

**Table 2:** List of plasmids.

Name	Details	Remarks, Reference
P1408	BS, *ime1*Δ::*hisG-URA3-hisG*	*IME1* deletion is from −1118 to +946
YIp1875	pBR322, *URA3*, *ime1*(-3.2 to +200)-*lacZ*	
P1583	BS, *ume6::hisG-URA3-hisG*	*UME6* is disrupted at aa 158
YIp2102	pBR322, *LEU2*, *IME1-UASru-his4-LacZ*	[28]
YEp2149	pBR322, *TRP1*, 2μ, *pADH1-GAL4(dbd*)	[37]
YIp2218	pBR322, *LEU2*, *GAL1uas-HIS4uas-his4-lacZ*	[37]
YIp2566	BS, *LEU2*, *rpd3::HIS3*	RPD3 deletion is from +98 to +744
YEp2593	pBR322, *TRP1*, 2μ, *ADHp-GAL4(dbd*)*-Rpd3-ADHt*	
P3109	T-easy, *rco1*Δ::*URA3*	*RCO1* deletion from −146 to +2498
P3144	pUC18, *sds3*Δ::*HIS3*	*SDS3* complete ORF deletion
P3215	T-easy, *dep1*Δ::*hisG-URA3-hisG*	*DEP1* deletion from −216 to +1365
P3153	T-easy, *sum1*Δ::*URA3*	*SUM1* deletion is from −1 to +3189
YIp3158	T easy, *rad17*Δ::*URA3*	complete deletion of ORF
YIp3179	*rco1*(+876 to +2051)-*6H* - *k1TRP1*	
YIp3182	*sds3*(+471 to +963)-13myc-tADH1, *HIS3*	
YIp3315	*RPD3*(-350 to +), *TRP1*	
YIp3316	*rpd3H150AH151, TRP1*	PEN153 (F. Posas) derivative

### Media and Molecular Genetic Techniques

SA (PSP2) and SPM media were prepared as reported previously [33]. Meiosis was induced as follows: Cells grown to early exponential stage (0.8–1.23 × 10^7^ cells/ml) in SA medium supplemented with the required amino acids were washed once with water and resuspended in SPM. β-Galactosidase assay: Proteins were extracted from 30 ml cells (1x10^7^ cells/ml) as described [34], and assayed for β-Galactosidase activity as described [35] . β-gal in Miller units were calculated per mg protein. Protein was measured using Bio-Rad Bradford kit. Staining with 4’,6-diamidino-2-phenylindole (DAPI) [36], and repression assays [37] were performed as described previously.

### Fluorescence-activated Cell Sorting (FACS)

Cellular DNA content was determined using FACS as described previously [38] using a FACScan analyzer (BD Biosciences, San Jose, CA). The percentage of cells with 4C DNA content was calculated using the WinMDI program (Joe Trotter, The Scripps Research Institute, La Jolla, CA, USA).

### Quantitative Analysis of RNA Expression

RNA was extracted from 1 × 10^8^ cells using the hot acidic-phenol method [39]. One microgram of total RNA was used as template for the reverse transcription reaction (total 20 µl) with random hexamer primers and SuperScript Reverse-iT transcriptase. Five nanograms of the cDNA product served as template for real-time polymerase chain reaction (qPCR) analysis according to the manufacturer’s instructions (ABGene, Surrey, UK).Primers used are: IME1: CAGCTGCAGAACTTGGTTCA and GTGGAACGTAGATGCGGATT (199 to 438); IME2: TAGACGCAAGAGGCAATGTG and ATCGTGATCGTTGTTGCTGA (1181 to 1341; NDT80: CTCGTCAATCCACACCAATG and CGGTTTCAGTTCGATTTGCT (1184 to 1428); NDT80AS: AAATGGAGGGCAATTATAAGG and CCTTGAATATACATAGTGTTTC (356 to -85); SUM1: TCTACGACCTCTGCGACAAT and CCGTCATCAAGGAAGTCAAA (2981 to 3114); TAF10: ATATTCCAGGATCAGGTCTTCCGTAGC and GTAGTCTTCTCATTCTGTTGATGTTGTTGTTG (390 to 530). 

*SUM1* and *TAF10* RNAs were used as controls because the transcription of both genes does not fluctuate in meiosis (http://derisilab7.ucsf.edu:591/public_spo/FMPro? and [40], respectively). 

### ChIP

 ChIP assays were performed essentially as described [37]. Following IP, genomic DNA was analyzed using qPCR (qChIP). Primers used are: IME2: CCAGCACTTGTCTGTGGCTT and CTGAGTGGCACAGCTTTTCC (amplicon -446 to -663); NDT80: CGCTCCAAGCTGACATAAAT and ATAGCCGCGGAAGTAACAA (amplicon -275 to -496); TEL1: GCG TAA CAA AGC CAT AAT GCC TCC and CTC GTT AGG ATC ACG TTC GAA TCC. Antibodies used are: Mouse monoclonal antibodies directed against c-myc (Ab-1, clone 9E11) was purchased from NeoMarkers. Ac-Histone H4 mouse monoclonal Antibody (Ser 1/Lys 5/Lys 8/Lys 12) G-2 was purchased from Santa cruze. 30 ml of cells (1x10^7^ cells/ml were IP with 4 µg/IP and 2µg/IP, respectively antibodies. 

## Results

### The Role of Rpd3L- and Rpd3S-Specific Components in Transcriptional Repression when Rpd3 is Ectopically Recruited to a Reporter Gene

**Figure 1:**
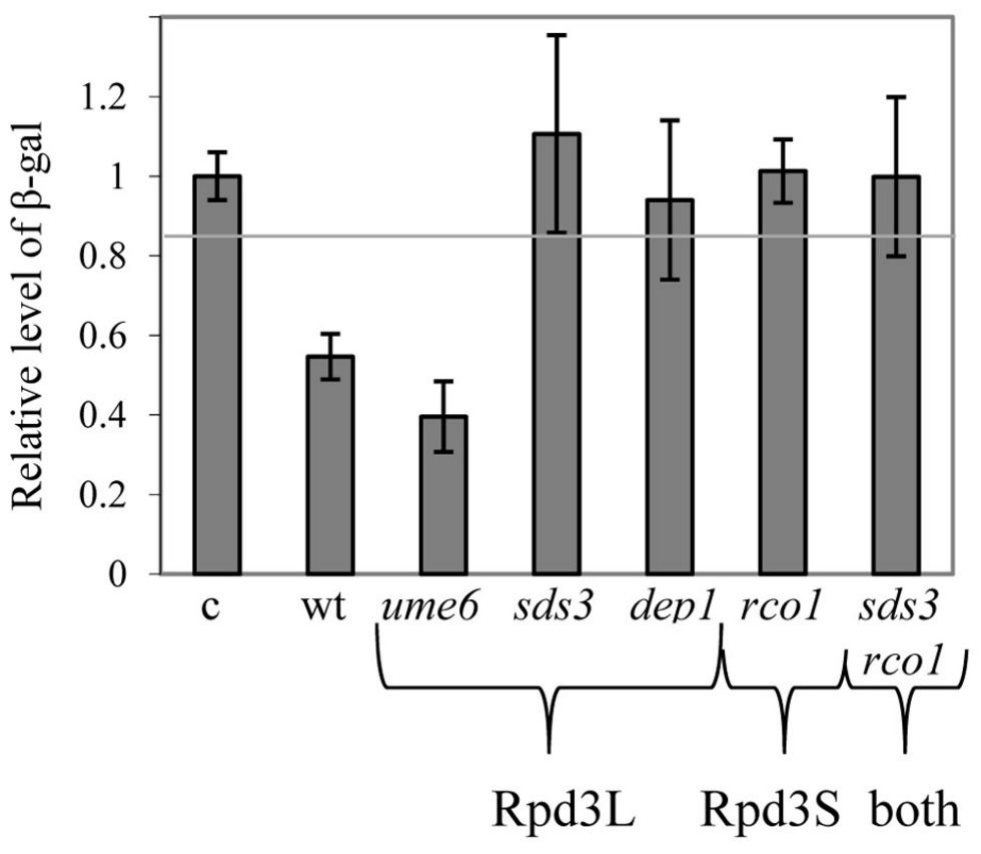
Repression of transcription by Rpd3 requires Rpd3L and Rpd3S. Cells carrying the *UAS*_*GAL1*_ *-*UAS_*HIS4*_ *-his4-lacZ* reporter gene were grown in SD medium to a density of 10^7^ cells/ml. The activity of β-galactosidase (Miller units) in cells expressing Gal4(dbd)-Rpd3 is relative to the level in the control cells (c) expressing only Gal4(dbd). Diploid strains used were as follows: wild-type (Y1884), *rco1*Δ/*rco1*Δ (Y1814), *sds3*Δ/*sds3*Δ (Y1845), *dep1*Δ/*dep1*Δ (Y1894), *ume6*Δ/*ume6*Δ (Y1388), and *rco1*Δ/*rco1*Δ *sds3*Δ/*sds3*Δ (Y1883). These strains carried either *pADH1-gal4*(dbd)*-*RPD3 (YEp2593) or *pADH1-gal4*(dbd) (YEp2149) on a 2-µ vector. Proteins were extracted from at least three independent transformants.

Repression assays were used to establish that Rpd3 represses transcription [17,37]. We useed this assay to determine if this function of Rpd3 depends on its presence in the large, small or both complexes. We asked whether Rpd3L and Rpd3S contribute to the ability of Rpd3 to repress transcription or possess distinct functions. For that purpose we used a repression assay. We generated diploid strains from which we deleted the genes as follows: *SDS3* or *DEP1* (two specific components of the large complex required for its integrity and activity [41]), *UME6* (an Rpd3L component required for targeting promoters of early meiosis-specific genes [13]), *RCO1* (an indispensable Rpd3S component required for activity [21,22]), or *SDS3* and *RCOI* (double mutant). The expression of a *UAS*_*GAL1*_ *-UAS*_*HIS4*_ *-his4-lacZ* reporter was compared between cells expressing either Gal4(dbd)-Rpd3 or Gal4(dbd) (dbd = DNA Binding Domain), when grown in SD (glucose) medium. Expression of Gal4(dbd)-Rpd3 in the wild-type strain reduced the level of expression of the reporter gene by a factor of two (Figure 1). A similar reduction was observed for *UME6* deletion mutant (Figure 1). These data suggest that Ume6 is not required for Rpd3 activity, consistent with its role in recruiting Rpd3L to DNA [13,17]. However, in isogenic diploid strains with deletions of *SDS3*, *DEP1*, *RCO1*, or an *SDS3-RCO1*-double deletion, repression by Rpd3 was not detected (Figure 1). These results suggest that Rpd3L and Rpd3S are both required for Rpd3 to repress transcription in cells grown in glucose-containing media.

### During Vegetative Growth with Acetate as the Sole Carbon Source, Rpd3L Represses Transcription, whereas Rpd3S Activates the Transcription of Meiosis-Specific Genes

The requirement for both Rpd3L and Rpd3S to repress transcription of the artificial reporter gene (*UAS*_*GAL1*_ *-UAS*_*HIS4*_ *-his4-lacZ*) in SD medium implies that they regulate meiosis-specific genes using the same mechanism. However, it is also possible that under physiological conditions these complexes possess specific functions determined by interaction with specific proteins present on the promoters of target genes that induce specific effects. Therefore, we determined the role of Rpd3L and Rpd3S complexes in regulating the transcription of genes that encode components of meiotic networks.

During vegetative growth with glucose as the sole carbon source, and independent of Rpd3, *IME1* is not transcribed [16,42,43]. Therefore, we assessed the effect(s) of Rpd3L and Rpd3S on vegetative growth with acetate as the sole carbon source (SA medium). Deletion of *RPD3* caused a significant increase in *IME1* transcription (Table 3), suggesting that Rpd3 represses *IME1* transcription. Deletion of either *SDS3* or *DEP1* caused a comparable increase in transcription, indicating that repression was mediated via Rpd3L (Table 3). Because deletion of *RCO1* led to a significant reduction in the transcription of *IME1* (Table 3), this suggests that Rpd3S functions as a positive regulator. Further, Rpd3 is recruited to the *IME1* promoter [16], suggesting that Rpd3S and/or Rpd3L exert a direct effect on the transcription of *IME1*. When both complexes were disrupted (*rco1*Δ *sds3*Δ-double mutant) (Table 3), the transcription of *IME1* was reduced, suggesting that the increase in the transcription of *IME1* in the *sds3*Δ strain depended on Rco1. Moreover, this reduced transcription was unexpected, because an opposite effect, namely transcription was increased in the *rpd3*Δ strain (Table 3). This result suggests that Rpd3 represses the transcription of *IME1* through an additional mechanism, which is independent of its presence and/or activity in Rpd3S or Rpd3L.

**Table 3:** Regulation of *IME1*, *IME2*, and *NDT80* transcription by Rpd3S and Rpd3L during vegetative growth.

	Relative level of RNA		
Strains	*IME1*	*IME2*	*NDT80*
wt	1.0 ±0.689	1.0 ±0.186	1.0 ±0.180
*rpd3∆*	32.509 ±0.184	18.390 ±0.662	1.070 ±0.445
*sds3∆*	17.768 ±2.102	8.0571 ±2.440	4.267 ±0.740
*dep1∆*	12.409 ±0.090	15.034 ±0.886	1.841 ±0.591
*sds3∆ rco1∆*	0.151 ±0.030	3.401 ±0.447	0.426 ±0.094
*rco1∆*	0.014 ±0.006	0.108 ±0.023	0.299 ±0.070
*rpd3H150AH151A*	0.336 ±0.317	1.937 ±0.322	0.359 ±0.157

RNA was purified from logarithmic cells grown to a density of 1x107 cells/ml in SA medium. Isogenic strains as follows: wt (Y1631), rco1∆/rco1∆ (Y1814), sds3∆/sds3∆ (Y1845), dep1Δdep1Δ (Y1894), and rco1∆/rco1∆ sds3∆/sds3∆ (Y1883), rpd3Δ/ rpd3Δ (Y1888), rpd3Δ/ rpd3Δ trp1Δ::TRP1-RPD3/trp1Δ::TRP1-RPD3 (Y2059), and rpd3Δ/ rpd3Δ trp1Δ::TRP1-rpd3H150AH151A/trp1Δ::TRP1-rpd3H150AH151A (Y2062) diploids. IME1, IME2, and NDT80 mRNA levels were determined using q-RT PCR and are expressed relative to those of either SUM1 or TAF10 (for strains Y2059 and Y2062). The relative level of RNA in each mutant in comparison to the wild-type strain is drawn. Results are the average value of three independent experiments, and standard deviation is included.

**Figure 2:**
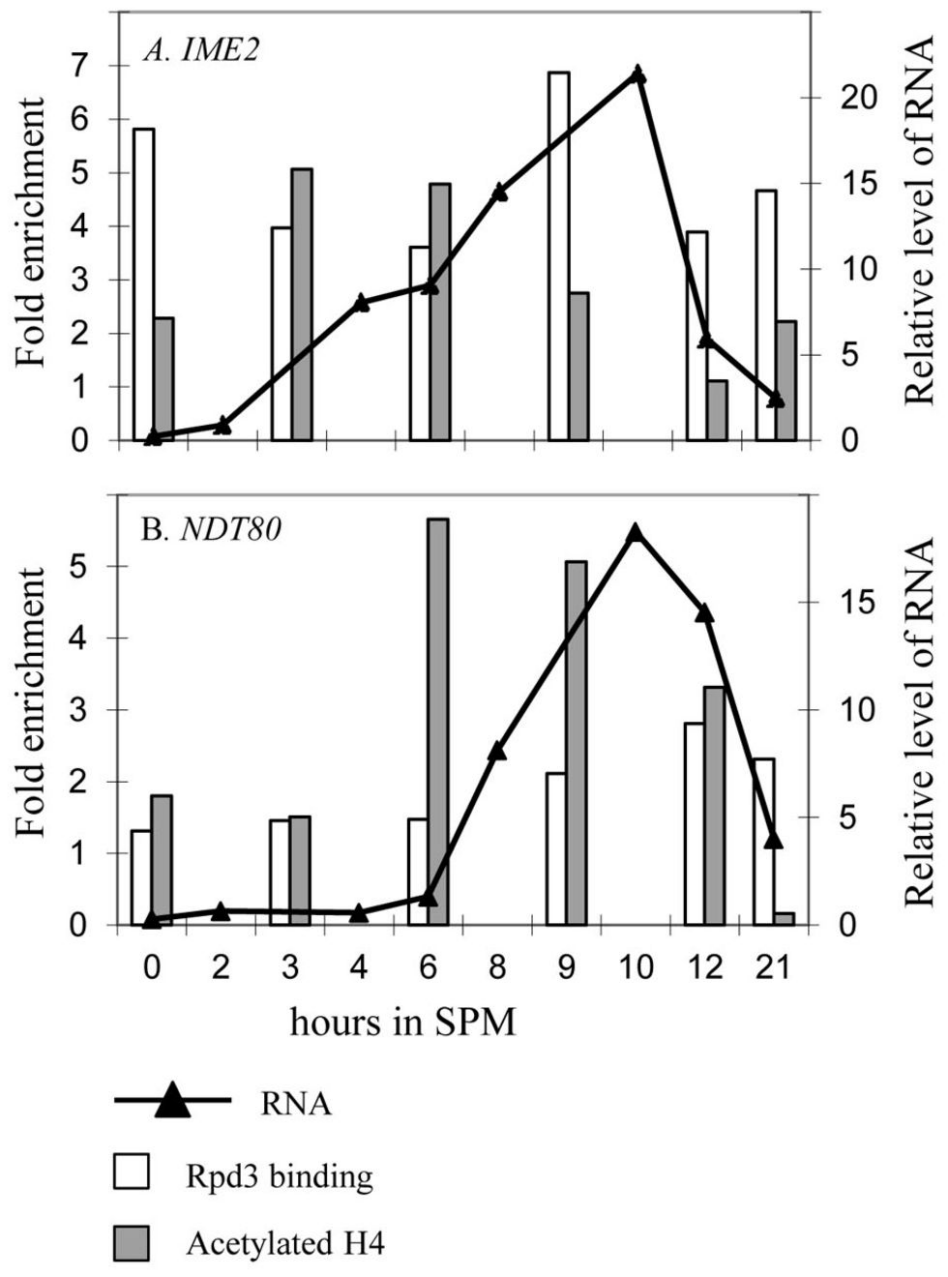
The kinetics of Rpd3 binding, histone H4 acetylation, and transcription of meiosis-specific genes. *MAT*a/*MAT*α *RPD3-13xmyc*/*RPD3-13xmyc* (Y1767) cells were shifted to meiotic conditions (SPM media) for the times indicated and subjected to ChIP analysis to determine Rpd3 binding (white column) and acetylated H4 (gray column). Sequences of the *IME2* (A), and *NDT80* promoters (B), or the *TEL1* locus were amplified using qPCR. Enrichment values represent the ratio between the relative levels of PCR amplicons recovered from the specific versus the non-specific probe, then the bound fraction was divided by input. Samples were taken simultaneously to isolate RNA for qPCR analysis (black line with triangles).

The effect on the level of transcription of *IME2* was similar to that observed for *IME1*, namely, repression by Rpd3L and activation by Rpd3S. Thus, transcription was increased in the *rpd3*Δ, *sds3*Δ, and *dep1*Δ strains (Table 3), and was reduced in the *rco1*Δ strain (Table 3). Because the transcription of *IME2* absolutely depends on Ime1 [44], these results indicate that the effect of Rpd3L and Rpd3S on *IME2* is indirect and is mediated via their effect on *IME1*. However, the *rco1*Δ *sds3*Δ-double mutant exhibited a specific phenotype, that is, the transcription of *IME2* increased whereas that of *IME1* was reduced (Table 3), suggesting that the effect of Rpd3 on *IME2* transcription is also direct. Moreover, the results of ChIP assays revealed that Rpd3 binds the *IME2* promoter in SA medium (Figure 2, time 0).

The transcription of *NDT80* in SA medium was increased in cells with deletions of either *SDS3* or *DEP1* (Table 3), suggesting that Rpd3L functions as a negative regulator of *NDT80* transcription. In contrast, deletion of a gene encoding an Rpd3S component, *RCO1*, reduced transcription, suggesting that Rpd3S functions as a positive regulator. Deletion of *SDS3* along with *RCO1* reduced transcription similarly, suggesting that the increase in transcription of *NDT80* by the *sds3*Δ mutant depends on Rco1. Deletion of *RPD3* had no detectable effect (Table 3), suggesting that the negative and positive effects of Rpd3L and Rpd3S counteracted each other, leading to no net effect. Moreover, the data also suggest that Rpd3 functions independently of Rco1-Sds3.

Table 4 summarizes these results, which show during vegetative growth with acetate as the sole carbon source, Rpd3L inhibits transcription of *IME1*, *IME2*, and *NDT80*, whereas Rpd3S activates their transcription. These findings are consistent with our hypothesis stated above regarding the specific functions of these complexes.

**Table 4:** The effects on transcription of Rpd3L, Rpd3S, and the noncatalytic activity of Rpd3.

	Catalytic Rpd3S			Catalytic Rpd3L		Non-catalytic	
media	positive		negative	positive	negative	positive	negative
**SA**		*IME1, IME2, NDT80*	-	-	*IME1, IME2, NDT80*	-	*IME1, IME2*
**SPM, early**		*IME1,IME2*		*IME1, IME2, NDT80*			*IME2*
**SPM, late**							*IME1, IME2, NDT80*

**Figure 3:**
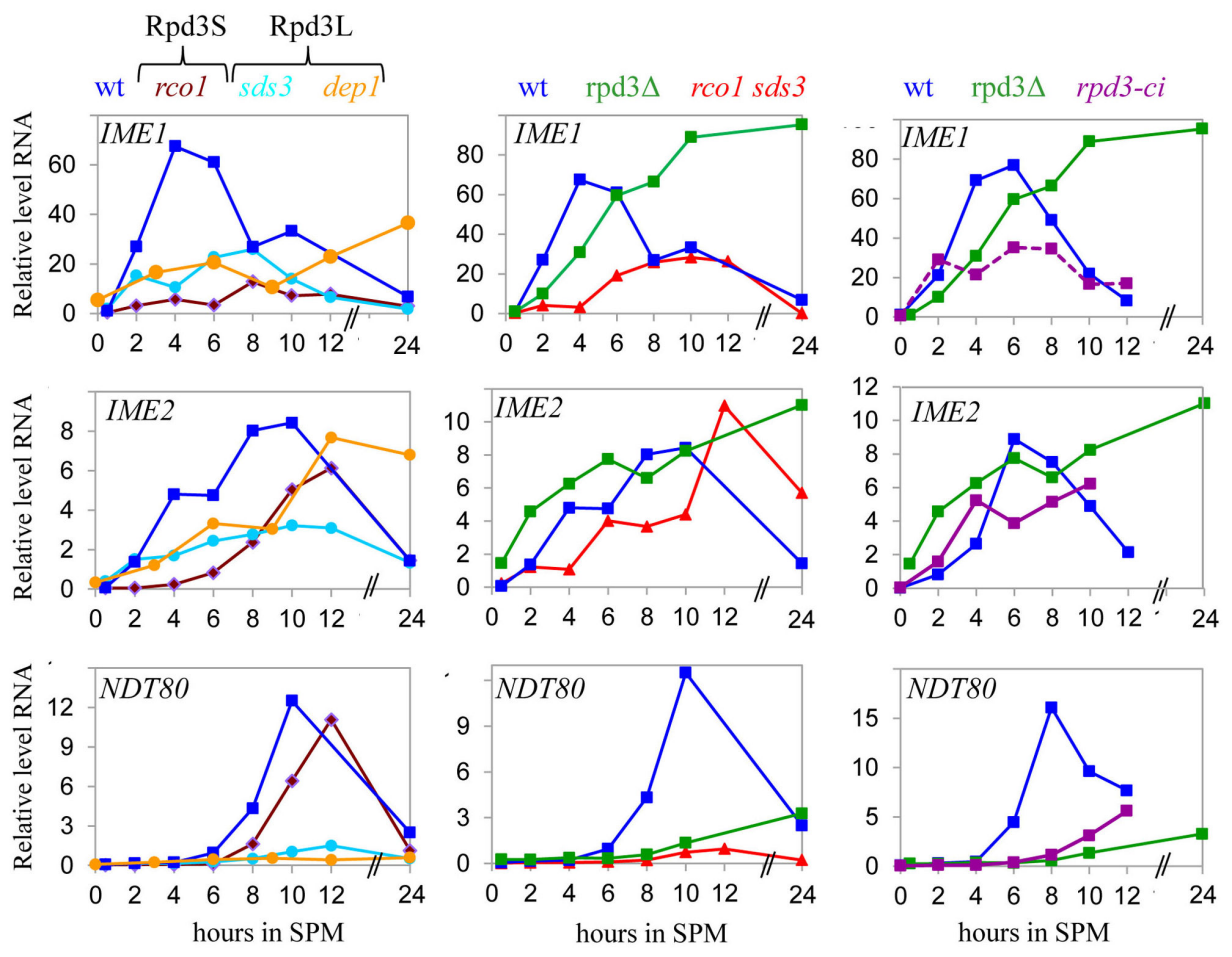
The effect of Rpd3L and Rpd3S mutations on the transcription of meiosis-specific genes. RNA was purified from cells grown to a density of 1x10^7^ cells/ml in SA and transferred to SPM for the indicated times. Isogenic strains were as follows: wt (Y1631), *rpd3*Δ/*rpd3*Δ (Y1888), *rco1∆/rco1∆* (Y1814), *sds3∆/sds3∆* (Y1845), *dep1∆/dep1∆* (Y1894), *rco1∆/rco1∆* *sds3∆/sds3∆* (Y1883) *rpd3*Δ/ *rpd3*Δ (Y1888), *rpd3*Δ/ *rpd3*Δ *trp1*Δ::*TRP1-RPD3*/*trp1*Δ::*TRP1-RPD3* (Y2059), and *rpd3*Δ/ *rpd3*Δ *trp1*Δ::*TRP1-rpd3H150AH151A*/*trp1*Δ::*TRP1-rpd3H150AH151A* (Y2062) diploids. The levels of expression of *IME1* *IME2* and *NDT80* were determined using q-RT PCR and are expressed relative to those of either *SUM1* or *TAF10* (for strains Y2059 and Y2062). The levels shown here are relative to the level of wt at time 0. The results shown are from a representative experiment. Similar results were obtained for at least three independent experiments.

**Figure 4:**
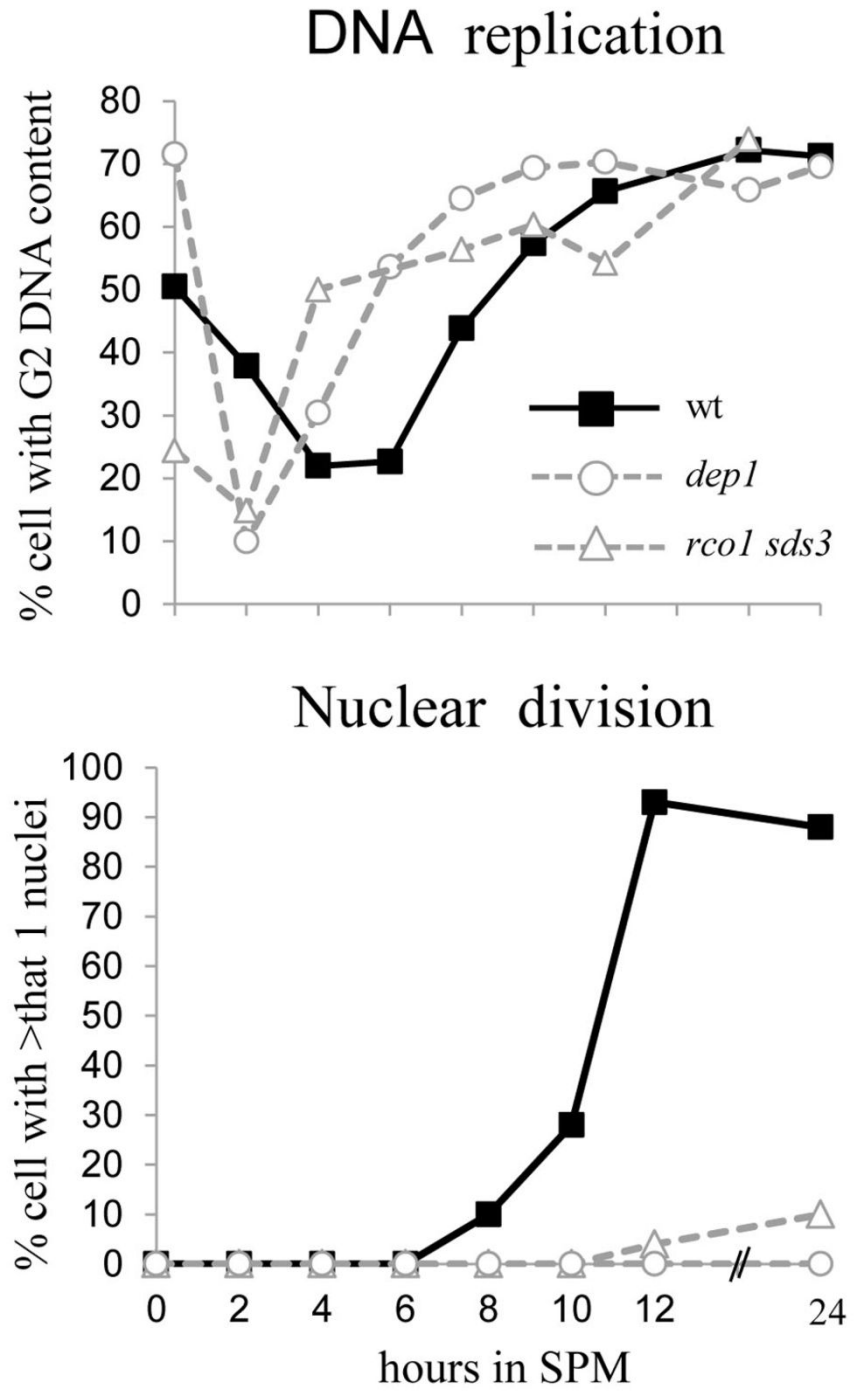
Diploids with *DEP1/RCO1* or *SDS3/RCO1* deletions arrest in meiosis before nuclear division. Isogenic wt (Y1631, closed squares), *dep1∆/dep1∆* (Y1894, open circles), and *rco1∆/rco1∆* *sds3∆/sds3∆* (Y1883, open triangle) diploids were shifted to meiotic conditions (SPM medium). Samples were taken at the indicated times for FACS analysis to calculate the percentage of cells with 4C DNA content and to count the percentage of cells with more than 2 nuclei (DAPI stain).

Summary of results: A positive and negative role for the catalytic activity of Rpd3S was assigned when the deletion of *RCO1* alone and together with *SDS3* resulted in a decrease and increase, respectively, in transcription (Figures 2 and 4). A positive and negative role for the catalytic activity of Rpd3L was assigned when the deletion of *SDS3* alone and together with *RCO1* resulted in a decrease and increase, respectively, in transcription (Figures 2 and 4). A positive and negative roles for the non-catalytic activity of Rpd3 was assigned when the deletion of *RPD3* resulted in an opposite result from *rco1 sds3* and *rpd3H150AH151A* mutants (Table 3 and Figure 3).

### The Role of Rpd3L and Rpd3S in the Expression of Meiosis-specific Genes during Meiosis

We examined the patterns of transcription of *IME1, IME2* and *NDT80* during meiosis of mutants with deletions of Rpd3L and Rpd3S components described above. Rpd3S and Rpd3L activate *IME1* transcription throughout meiosis. Deletion of either *RCO1* or *SDS3* reduced *IME1* transcription, albeit the effect of the absence of Rco1 was greater (Figure 3). The pattern of *IME1* expression of the *rco1*Δ *sds3*Δ double mutant suggests that at early meiotic times the effect was mediated mainly by Rpd3S (Rco1) while at later times by Rpd3L (Sds3). Moreover, the epistatic relationship between *RCO1* and *SDS3* (no additive effect when both genes were deleted) suggests that these complexes deacetylate the same substrate. We expected that deleting *DEP1*, which is a specific component of Rpd3L, would exhibit the same phenotype as deleting *SDS3*, and that deletion of *RPD3* will give the same phenotype as deletion of both *RCO1* and *SDS3*, namely reduced transcription. However, this was not the case, because early in meiosis, Dep1 and Rpd3 functioned as positive regulators, but at later times as negative regulators (Figure 4), suggesting that Dep1 and Rpd3 may possess additional functions not in common with Sds3 and Rco1.

The transcription of *IME2* was increased throughout meiosis in a strain deleted for *RPD3* (Figure 3), suggesting that Rpd3 inhibits *IME2* transcription. We assumed that Rpd3L mediates repression, because Rpd3 is recruited to *IME2* by Ume6, which is a component of this complex [13,19]. However, to our surprise, deletion of *SDS3* decreased transcription rather than increasing it (Figure 3). This reduction in transcription might be indirect through reduced *IME1* transcription, because the level of transcription of *IME2* responds in a gradient mode to Ime1 levels [45]. A different picture emerged when we examined the effect of the Rpd3L component Dep1. Deletion of *DEP1* reduced *IME2* transcription similarly to that of the *sds3*Δ strain. However, during late meiosis, transcription increased, similar to that of the *RPD3* deletion mutant (Figure 3).

*IME2* transcription in the *rco1*Δ diploid did not resemble that of the *rpd3*Δ strain (Figure 3). Moreover, it was not as robust as wild-type, because complete induction took significantly longer (Figure 3). Evidence indicates that the reduction in the level of *IME1* transcription immediately reduces that of *IME2* [45], suggesting that the positive effect of Rco1 (Rpd3S) on *IME2* transcription is direct. The transcription of *IME2* in the *rco1*Δ *sds3*Δ-double mutant resembled the *rco1*Δ pattern but differed from that of *rpd3*Δ (Figure 3), suggesting that Rpd3 repressed the transcription of *IME2* independent of Rpd3S and Rpd3L.

Next, we determined the effects of Rpd3L and Rpd3S on the transcription of *NDT80*. In diploid cells with deletions of *SDS3, DEP1*, or *RPD3* the usual induction of *NDT80* transcription was not detected (Figure 3). This result could not be attributed to the effect on *IME1* and *IME2* transcription, because this would only delay *NDT80* [45]. Therefore, we conclude that Rpd3L mediates activation of *NDT80* transcription. In contrast, in diploid cells with *RCO1* deletion, the transcription of *NDT80* was initiated with a delay, but it reached the same level as that of the isogenic wild-type strain (Figure 3). Because the transcription of *NDT80* is absolutely dependent on *IME2* [46], we suggest that the effect of Rco1 on *NDT80* is mediated through Ime2 either directly (Ime2 may recruit Rpd3S), or indirectly (the reduced level of Ime2 delayed the transcription of *NDT80*). 

The level and pattern of expression of *NDT80* in the *rco1*Δ *sds3*Δ-double mutant was similar to that observed for the *sds3*Δ or the *rpd3*Δ isogenic strains for 12 hours in SPM (Figure 3), suggesting that the positive effect of Rpd3 is mediated solely by Rpd3L. However, after 24 hours in SPM, transcription in the *RPD3* deletion mutant increased while in the *rco1*Δ *sds3*Δ mutant, the level of expression remained low (Figure 3). This result suggests that late in meiosis, the decline in the transcription of these genes requires Rpd3 but not Rco1 and Sds3. 

In summary (Table 4), under meiotic conditions, both Rpd3S and Rpd3L function as positive regulators of *IME1* and *IME2* transcription. In contrast, *NDT80* transcription is positively regulated only by Rpd3L. Rpd3 also functions as a negative regulator independent of its presence in the Rpd3L and Rpd3S complexes.

### Rpd3L is required for Meiotic Nuclear Division whereas Rpd3S does not Affect Meiosis

The above results indicate that Rpd3S functions as a nonessential positive regulator of *IME1* and *IME2* and plays no role in *NDT80* transcription (Figure 3). Because meiosis is robust and insensitive to the levels of its positive regulators (Ime1, Ime2, and Ndt80) [45], as expected, diploid *RCO1*-deletion mutants sporulated, producing 81.4% asci after 48 h culture in SPM. In contrast, deletion of specific components of the Rpd3L complex, *SDS3* and *DEP1*, reduced *IME1* and *IME2* transcription but *NDT80* was not transcribed (Figure 3). These results predict that diploid cells harboring mutations in Rpd3L components will arrest in the meiotic pathway following completion of premeiotic DNA replication but prior to nuclear division. Note that Ndt80 is required for the transcription of the middle meiosis-specific genes, which encode proteins required for nuclear division [47]. In agreement with this prediction, diploid cells with either *DEP1* or *SDS3* deletions along with *RCO1* deletions, completed premeiotic DNA replication and accumulated cells with a single nucleus (Figure 4).

### Transcriptional Repression and Catalysis by Rpd3 can Function Independently

The results described above demonstrate that Rpd3 represses transcription during the late stages of meiosis independent of either Sds3 or Rco1 (Table 3 and Figure 3). Similarly, using a lexA-Ume6 reporter it was reported that Rpd3 may repress transcription independent of its histone deacetylase activity [48]. Moreover, the mechanism of repression apparently involves nucleosome stabilization by the Rpd3 core complex [49]. We postulated that diploid cells expressing catalytically inactive Rpd3 would exhibit a different phenotype than cells with a deleted *RPD3* allele and phenotype similar to that of *sds3*Δ *rco1*Δ diploids. A catalytic inactive Rpd3 mutant was constructed by mutating His150 and His151 to Ala [48,49]. The mutant and wild-type (control) genes were each inserted into the *TRP1* loci of an *RPD3*-deletion mutant, and *IME1*, *IME2*, and *NDT80* transcription was measured during vegetative growth with acetate as the sole carbon source (Table 3) as well as during meiosis (Figure 3).

*IME1* transcription in the *rpd3H150AH151A* and *sds3*Δ *rco1*Δ strains was reduced but increased in the *rpd3*Δ diploid cultured in SA (Table 3), suggesting that as predicted, *IME1* transcription was repressed by a noncatalytic function of Rpd3. *IME1* transcription was reduced during late meiosis in the wild-type as well as strains carrying the *rpd3H150AH151A* allele or *sds3*Δ *rco1*Δ-double mutants. In contrast, *IME1* expression increased in the *rpd3*Δ strain (Figure 3), demonstrating that its transcription was independent of the catalytic activity of Rpd3.

*IME2* transcription was elevated in cells carrying the *rpd3H150AH151A* allele and grown in SA medium, similar to the increase observed in cells with *RPD3*, *SDS3, DEP1* deletions or *SDS3*/*RCO1*-double deletion (Table 3), implying that only the catalytic activity of Rpd3L repressed *IME2* transcription. Nonetheless, deletion of *RPD3* increased transcription by a factor of 18, in contrast to the two-fold increase in cells expressing the catalytically active mutant. This result suggests that the catalytic and noncatalytic functions of Rpd3 repress *IME2* transcription. At late meiotic times *IME2* transcription was decreased in cells that expressed the catalytically inactive Rpd3 allele, although it was elevated in cells lacking Rpd3 (*rpd3*Δ) (Figure 3). The results suggest that under this condition Rpd3 activity was noncatalytic, reinforcing the conclusion that at this time, repression by Rpd3 was independent of its presence in Rpd3L or Rpd3S.

Expression of a catalytically inactive Rpd3 mutants cultured in SA medium reduced *NDT80* transcription similar to the level of the *rco1*Δ strain (Table 3), implying that the catalytic activity of Rpd3 in Rdp3S activates *NDT80* transcription. During early meiosis, the phenotypes of the *rpd3H150AH151A* and *rpd3*Δ strains were similar (Figure 3), indicating that only the catalytic activity of Rpd3 was required for transcriptional activation of *NDT80*. However, transcription increased in the mutant carrying the point mutations in comparison to the wild-type strain (Figure 3), suggesting that transcriptional repression was mediated by the noncatalytic function of Rpd3.

Therefore, our results validate the prediction that Rpd3 functions catalytically as well as noncatalytically. The catalytic activity exerted either positive or negative effects, whereas the noncatalytic activity only repressed transcription (Table 4).

### *NDT80* Transcription Correlates with Histone H4 Acetylation

Deacetylation of lysine residues in the N-terminal tail domain of histones H3 and H4 is associated with either transcriptional repression or activation [1-4]. Therefore, using qChIP assays, we asked whether *NDT80* transcription depends on deacetylation of histone H4. The mRNA level was determined simultaneously. We amplified *NDT80* and *IME2* as a control to represent early meiosis-specific genes.

*IME2* transcription correlated with reduced Rpd3 binding to *IME2* and a concomitant increase in histone H4 acetylation at the *IME2* promoter region (Figure 2A) in agreement with previous findings [50]. Moreover, the decline in transcription correlated with increased Rpd3 occupancy and decreased acetylation (Figure 2A). Acetylation and transcriptional activation of *NDT80* also correlated (Figure 2B). However, Rpd3 binding to the *NDT80* promoter was detected only during late meiosis when *NDT80* transcription was reduced (Figure 2B), suggesting that the effect of Rpd3 on the transcription of *NDT80* was not mediated by deacetylation of histone H4.

### Possible Mechanisms of Transcriptional Activation by Rpd3

#### The pachytene checkpoint

**Figure 5:**
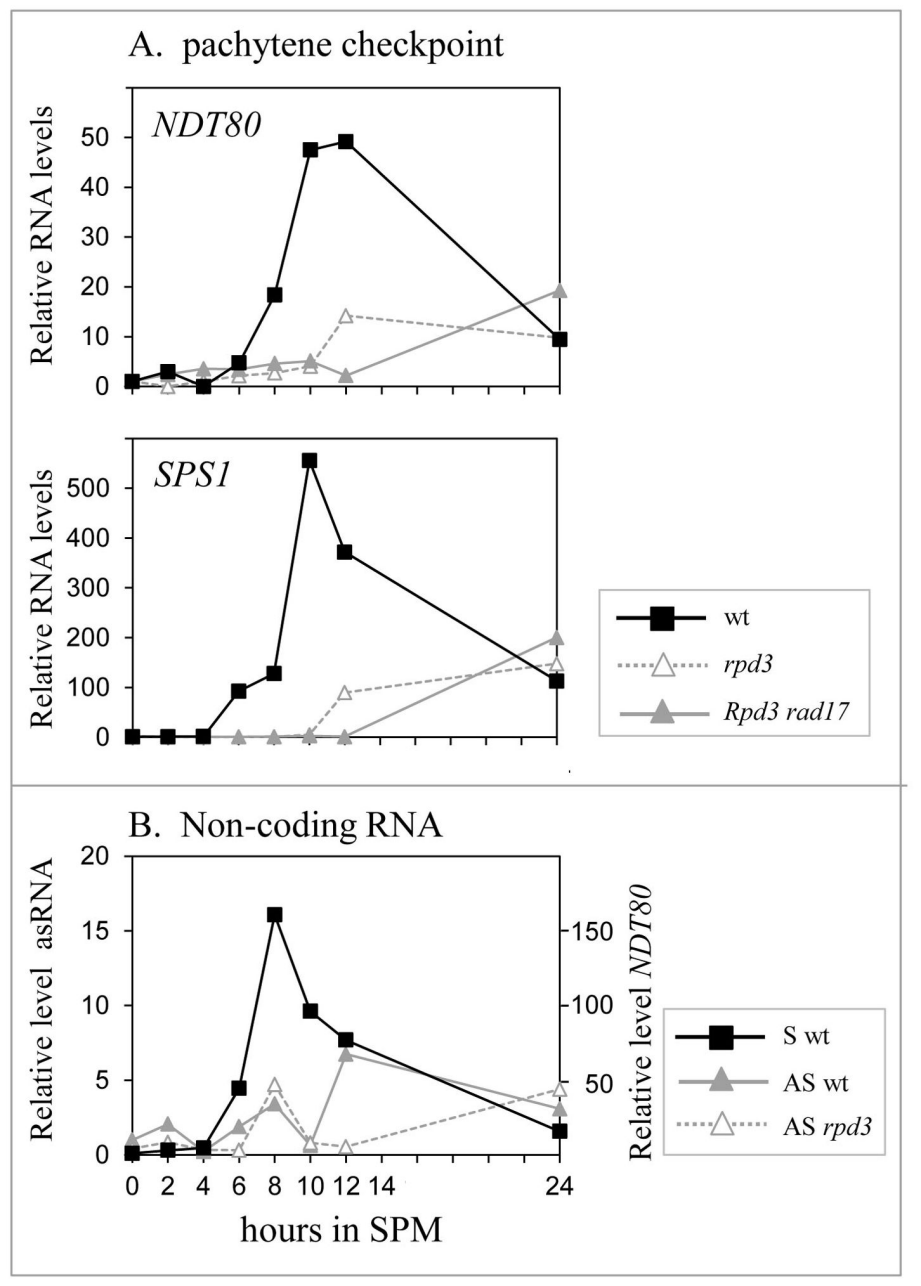
Possible molecular mechanisms of transcriptional activation by Rpd3. A. Rpd3 does not activate transcription through the pachytene checkpoint. Isogenic strains used were as follows: (Y1631, black squares), *rpd3*Δ::*HIS3*/*rpd3*Δ::*HIS3* (Y1537, empty gray triangle, dashed gray lines), (*rpd3*Δ::*HIS3*/*rpd3*Δ::*HIS3*, *rad17*Δ::*URA3*/*rad17*Δ::*URA3* (Y1848, gray triangle, gray lines). B. *NDT80* antisense RNA does not mediate transcriptional activation of *NDT80* by Rpd3. Strains used were as follows: wild-type (Y2059) and *rpd3*Δ::*HIS3*/*rpd3*Δ::*HIS3* (Y1888). Relative level of *NDT80* RNA in the wild-type strain (S, black square, black line). Relative levels of *NDT80* antisense RNA (AS) in the wild-type strain (gray triangle, gray line) and *rpd3*Δ strain (empty gray triangle, dashed line).

Cells impaired in meiotic recombination arrest in meiosis before *NDT80* transcription commences [32], an arrest that depends on the pachytene checkpoint pathway (for review see 51). Because Rpd3 is required for the appropriate response of the ATR checkpoint to double-strand DNA breaks [52], we postulated that Rpd3 is required to relieve the inhibition mediated by the checkpoint during the recombination process, to promote the *NDT80* transcription. This hypothesis predicts that deletion of *RAD17* (a checkpoint component) would suppress *rpd3*Δ and promote *NDT80* transcription. This hypothesis was discarded, because *rpd3*Δ/*rpd3*Δ *rad17*Δ/*rad17*Δ diploids remained sporulation deficient and did not express *NDT80* or *SPS1* (a mid-meiosis-specific gene) (Figure 5A).

#### Noncoding RNA

Numerous noncoding sense and antisense RNAs are transcribed from the yeast genome [53-55]. These RNAs might interfere with the transcription of coding RNA as reported, for example for the meiosis-specific genes *IME4* [56] and *IME1* [57]. Because the *NDT80* promoter drives the transcription of an antisense RNA (–78 to –390, http://yeast.utgenome.org/ and [53]), we postulated that this RNA inhibits transcription of coding sequences. Moreover, we suggested that Rpd3 is required to repress the transcription of this *NDT80*-antisense RNA and consequently promotes the transcription of *NDT80*. However, this hypothesis was disproved, because the levels of this antisense transcript were similar in wild-type and *rpd3*Δ diploids (Figure 5B). Moreover, the transcription of this antisense RNA was induced at the same time as the coding RNA (Figure 5B). 

#### Deacetylation of nonhistone proteins

**Figure 6:**
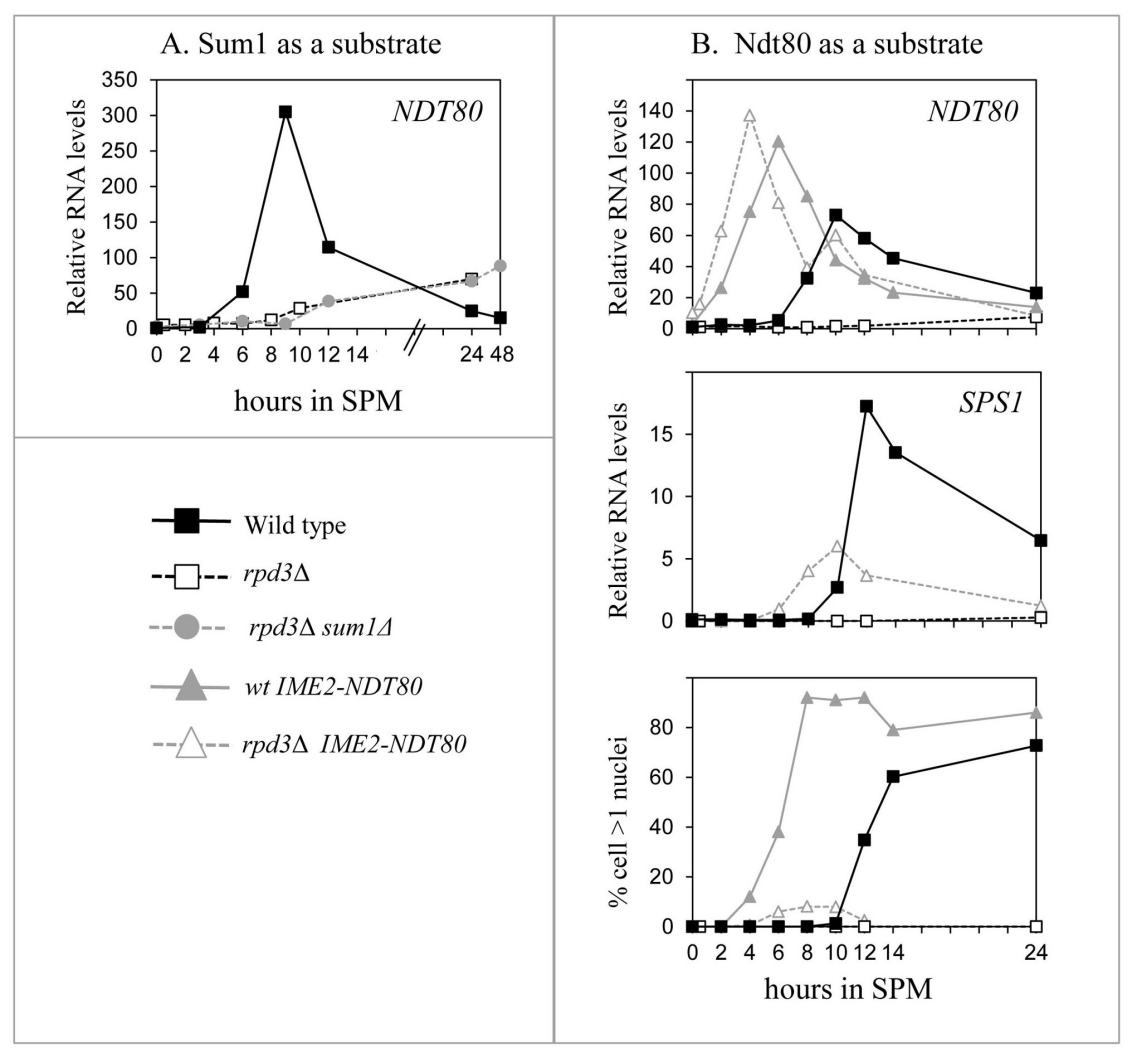
Possible molecular mechanisms of transcriptional activation by Rpd3. A. Deletion of *SUM1* did not suppress *rpd3*Δ. Isogenic wild-type (Y1631, closed black squares), *rpd3*Δ/*rpd3*Δ (Y1888, empty black squares, dashed line) and *rpd3*Δ/*rpd3*Δ *sum1*Δ*/sum1*Δ (Y1914, gray circles, dashed lines). B. Ectopic transcription of *NDT80* partially suppressed the effect of *rpd3*Δ on the transcription of *SPS1* and nuclear division. Isogenic *NDT80*/*NDT80* (Y1631, black squares), *IME2p-6xHA-NDT80/IME2p-6xHA-NDT80* (Y1763, gray triangles), *rpd3*Δ/*rpd3*Δ (Y1537, empty gray squares, dashed gray lines) and *rpd3*Δ/*rpd3*Δ *IME2p-6xHA-NDT80/IME2p-6xHA-NDT80* (Y1870, empty gray triangle, dashed gray lines) cells were shifted to meiotic conditions (SPM medium), and at the indicated times, samples were taken for RNA extraction and DAPI staining to determine the percentage of cells with more than 1 nucleus. *NDT80* expression was measured using q-RT PCR. Levels of expression are relative to that of *ACT1*. The results of a representative experiment are shown. Similar results were obtained from three independent experiments.

Proteomic analysis reveals that many, nonhistone proteins are subjected to acetylation, which affects their function (for review see 58). Because *NDT80* transcription correlated with histone H4 acetylation rather than deacetylation (Figure 2B), and because it depended on the catalytic activity of Rpd3, we postulated that the positive effect of Rpd3 was mediated through a nonhistone protein. The transcription of *NDT80* is repressed by Sum1 and activated by Ime1, Ime2, and Ndt80 [59]. Therefore, we first asked whether Sum1 acetylation is required for its ability to repress *NDT80* transcription and whether its deacetylation by Rpd3 relieves this repression. This hypothesis predicts that deletion of *SUM1* will suppress *rpd3*Δ; however, a *sum1*Δ *rpd3*Δ diploid strain was sporulation deficient, and *NDT80* was not transcribed (Figure 6A). We conclude, therefore, that the effect of Rpd3 is not solely mediated by deacetylation of Sum1.

We next asked whether deacetylation of Ndt80 is required for its activity and consequently for transcription of mid-meiosis-specific genes and initiation of nuclear division. This hypothesis predicts that ectopic expression of Ndt80 will not suppress *rpd3*Δ; however, *NDT80* expression controlled by the *IME2* promoter led to a significant increase in the transcription of the mid-meiosis-specific gene *SPS1* (Figure 6B) as well as the accumulation of cells with two nuclei (Figure 6B). The effect was only partial, likely because ectopic expression of *NDT80* in the wild-type strain is deleterious; cells initiated premeiotic DNA replication and nuclear division simultaneously [45]. We conclude, therefore, that the effect of Rpd3 on the transcription of *NDT80* and mid-meiosis-specific genes is not mediated solely by an effect on the function of Ndt80.

## Discussion

The choice between alternative developmental pathways is mainly controlled at the level of transcription. Frequently, genes that are specific for one pathway are silenced in cells that are engaged in an alternative pathway. Gene inactivation can result from lack of specific activators and from active repression. Thus, during vegetative growth of budding yeasts, histone deacetylation mediated by Rpd3 silences EMGs [19,60]. During meiosis, the transcription of these genes requires two Ime1-dependent events, relief of repression, and transcriptional activation [37]. The effect of Rpd3 on meiosis is not mediated through its effect only on EMG, because it regulates the transcription of all meiosis-specific genes that differ in magnitude and stage. For example, Rpd3 functions as a positive regulator of *IME1* early in meiosis and as a negative regulator at later stages ([16] and Figure 3). *NDT80* transcription is similarly influenced, notwithstanding that Rpd3 is indispensable for *NDT80* transcription as well as mid- and late meiosis-specific genes ([32] and Figures 3, 5-6). 

**Figure 7:**
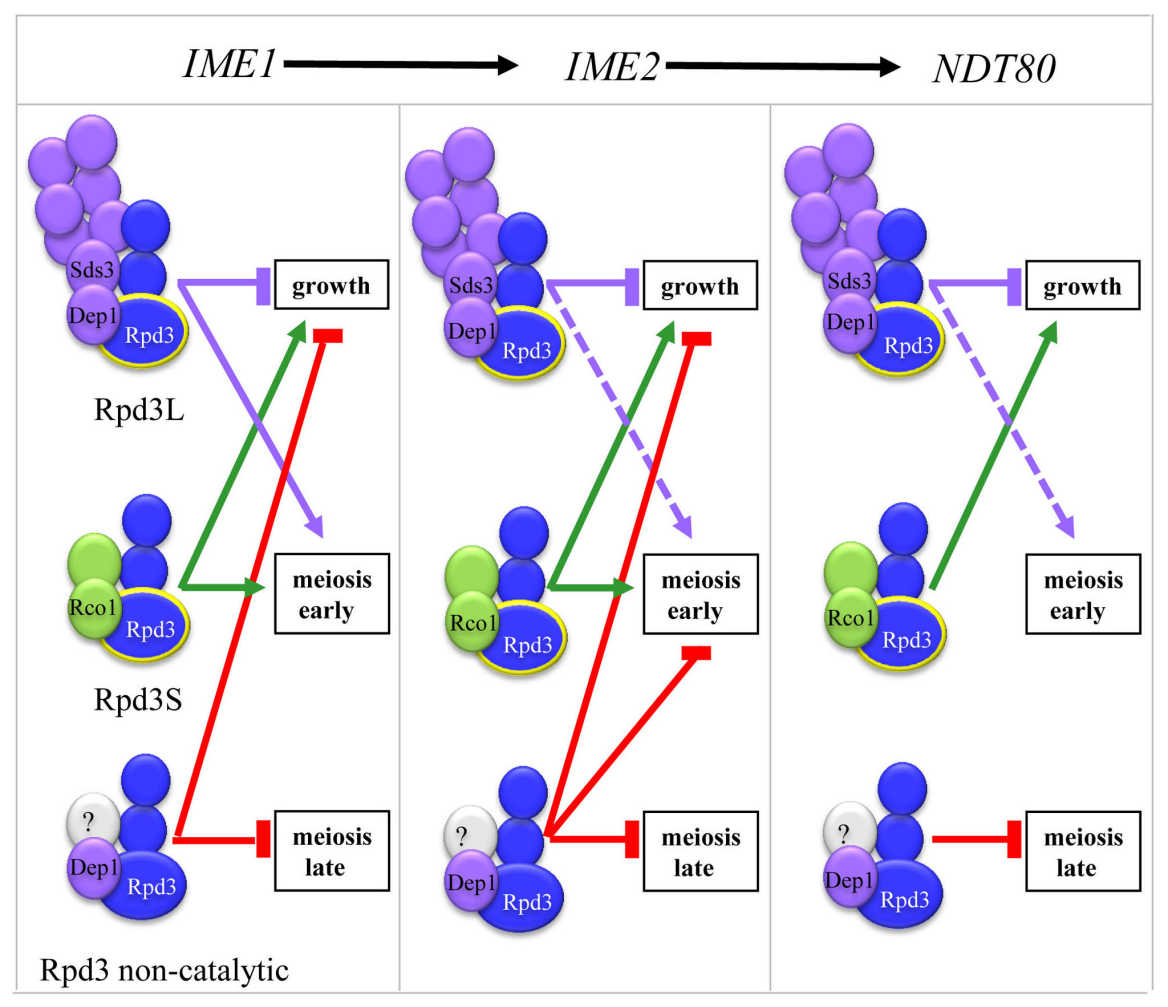
Model for the regulation of meiosis-specific genes by Rpd3. Blue rectangle: Core components; Purple rectangle: components specific to Rpd3L; Green rectangles components specific to Rpd3S. The components whose function was examined in this research are marked. Growth: Transcription under vegetative growth conditions with acetate as the sole carbon source. Meiosis: nitrogen depletion in the presence of acetate as the sole carbon source.

Our goal here was to determine the mechanisms underlying the dual and opposing functions of Rpd3. We reasoned that a likely explanation is the presence of Rpd3 in two distinct complexes that target specific genes.. To study the functions of Rpd3 in either Rpd3L or Rpd3S, we genetically deleted *SDS3* or *DEP1* from Rpd3L and *RCO1* from Rpd3S. We studied the effect of Rpd3 on the transcription of three major positive regulators of meiosis as follows: 1) *IME1*, the master transcriptional activator, is essential for the transcription of all meiosis-specific genes; 2) *IME2*, a representative of the network of early genes, is essential for the transcription of middle and late genes; and 3) *NDT80*, an early-middle gene, is essential for the transcription of middle and late genes (for review see 61). These genes respond in specific modes to Rpd3, and thus serve as a paradigm to study the requirements for Rpd3L and Rpd3S. The results show that Rpd3 has at least four distinct modes of action, depending on the gene(s) studied and growth conditions as follows: 1) Rpd3L and Rpd3S require Rpd3 to repress transcription. 2) Rpd3 functions only as a positive regulator in Rpd3S. 3) Rpd3 switches from a negative to a positive regulator depending on nitrogen depletion in Rpd3L. 4) the noncatalytic activity of Rpd3, which is independent of the integrity of Rpd3L and Rpd3S, represses transcription. Figure 7 summarizes the results for the control of the meiosis-specific genes *IME1*, *IME2* and *NDT80* by Rpd3. 

### The Integrity of both Rpd3S and Rpd3L is Required for Repression

Gal4(dbd)-Rpd3 repressed transcription of a synthetic gene, albeit repression depended on the integrity and function of Rpd3S and Rpd3L (Figure 1). This indicates that for a specific gene, repression depends on the activity of Rpd3 when it interacts with promoter and ORF sequences as suggested previously [21,22]. In SA media repression by Rpd3 is relieved [37]. Furthermore, after six hours in SPM, the β-gal levels of the reporter in strains expressing Gal4(dbd-Rpd3) or Gal4(dbd) were 10.32 ± 1.89 and 10.37 ± 1.57 Miller units, respectively, suggesting relief of repression under this condition. 

### Rpd3S Functions Specifically as a Positive Transcriptional Regulator

Transcription of the meiosis-specific genes *IME1*, *IME2*, and *NDT80* responded uniquely to Rpd3 present in Rpd3L and Rpd3S. Their expression was decreased in mutants that expressed only Rpd3L (Tables 3 and 4), whereas their expression was increased in mutants that expressed only Rpd3S (Tables 3 and 4). The ability of Rpd3S to activate transcription can be explained by the observation that the Eaf3 subunit of Rpd3S is also a subunit of the transcriptional activator NuA4-HAT [12]. Indeed, the opposing effects of Rpd3S and Rpd3L were revealed by specific genetic interactions with FACT or NuA4 [62]. In summary, our results support a model in which Rpd3L functions as a repressor, whereas Rpd3S activates transcription. 

Nitrogen depletion did not change the positive effect of Rpd3S on the transcription of *IME1* and *IME2* (Table 4 and Figure 3). However, *NDT80* transcription did not require Rpd3S, and Rpd3 activated transcription only as a component of Rpd3L (Table 4 and Figure 3). Accordingly, cells with *RCO1* deleted sporulated, whereas cells with *DEP1* or *SDS3* deletions along with *RCO1* arrested in meiosis and contained single nuclei after completing premeiotic DNA replication (Figure 4).

### Rpd3L Switches its Activity from a Negative to a Positive Regulator Depending on Nitrogen Depletion

As described above, in cells cultured in SA medium, Rpd3L negatively regulates transcription of *IME1*, *IME2*, and *NDT80*, consistent with findings that deletion of *UME6*, which is an integral component of this complex [13] derepresses transcription of EMGs [63]. However, in the absence of a nitrogen source during early and mid-meiosis, Rpd3L acts specifically to induce *IME1* and *NDT80* transcription (Figure 3 and Table 4). The effect of Rpd3L on the transcription of *NDT80* might be indirect, through its effect on the transcription of *IME1*, because Ime1 directly activates *NDT80* transcription. We rejected this hypothesis, because the reduced Ime1 level is expected to delay and attenuate the transcription of *NDT80*, rather than inhibit it [45] as revealed in strains deleted for either *SDS3* or *DEP1* (Figure 3).

The temporal switch in Rpd3L from a transcriptional repressor to an activator did not correlate with decreased histone acetylation. On the contrary, before either *IME2* or *NDT80* transcription was induced, the level of acetylated histone H4 increased (Figure 2), suggesting indirect activation by Rpd3L. Three possible mechanisms were examined: 1) Rpd3 is required to inhibit the pachytene checkpoint, which inhibits *NDT80* transcription in the presence of nicks/breaks in the DNA strands [51]. Thus, the absence of Rpd3 at this checkpoint inhibits *NDT80* transcription. We rejected this mechanism, because inactivation of the checkpoint by deleting *RAD17* did not suppress *rpd3*Δ (Figure 5A). 2) High-throughput RNA sequence analysis revealed that *IME1, IME2*, and *NDT80* express a noncoding RNA (http://yeast.utgenome.org). The noncoding RNA transcribed from the *IME1* promoter represses, through the Set3 histone deacetylase, *IME1* transcription in non-*MAT*a/*MAT*α strains [57]. This repression is independent of transcriptional activation by Rpd3, because it occurs in diploid cells that do not express this RNA. We did not examine the effect of antisense RNA on *IME2* transcription, because recruitment of Rpd3 to the *IME2* promoter by Ume6 induced deacetylation of histones H3 and H4, and consequently repression [17,48]. The *NDT80* promoter expresses an antisense RNA [53]. The possibility that Rpd3 inhibits transcription of the *NDT80* antisense RNA to induce transcription of the *NDT80* ORF was rejected, because the induction of *NDT80* transcription did not correlate with reduced levels of this RNA (Figure 5B). *NDT80* antisense RNA expression first peaked simultaneously with that of the *NDT80* ORF mRNA and was not affected by Rpd3. The presence of a second antisense peak depended on Rpd3 and correlated with decreased *NDT80* transcription (Figure 5B), indicating that the decline in *NDT80* transcription may be mediated through this RNA (3).. Acetylation regulates the activities of a wide array of nonhistone proteins as well as transcription factors [58,64]. These findings suggest that Rpd3 deacetylates transcription factors to modulate their activities. *NDT80* transcription depends on four transcriptional activators, Ime1, Ime2, Ndt80, and Rpd3 as well as on the negative regulator Sum1 (reviewed in 61) that may be regulated by acetylation. We examined the possibility that the inhibitory activity of Sum1 depends on its acetylation, which is reversed by its deacetylation by Rpd3. This hypothesis predicts that deletion of *SUM1* will suppress *rpd3*Δ. However, the *rpd3*Δ *sum1*Δ strain did not sporulate (Figure 6A), and the hypothesis was refuted. The second candidate examined was Ndt80. We postulated that acetylation of Ndt80 inhibits its ability to activate transcription. This model predicts that expression of Ndt80 from a heterologous promoter will not suppress *rpd3*Δ. However, *NDT80* expression from the *IME2* promoter in *rpd3*Δ cells promoted the transcription of the mid-meiosis-specific gene *SPS1*, and a fraction of cells initiated nuclear division (Figure 6B). Further work is therefore required to determine if Rpd3 deacetylates Ime1 and/or Ime2 or a different substrate(s).

### Noncatalytic Transcriptional Repression by Rpd3

In budding yeast, following gametogenesis, an additional developmental pathway takes place, spore formation. At this time, the transcription of all meiosis-specific genes decline. We attribute this to histone deacetylation, because deletion of *RPD3* caused persistent transcription (Figure 3) accompanied by reduced levels of histone H4 acetylation (Figure 2). We predicted therefore that deletion of *RCO1* along with *SDS3*, would abolish Rpd3 activity and generate the *rpd3*Δ phenotype. Surprisingly, the phenotype exhibited by the *rco1*Δ *sds3*Δ-double mutant was unique, late in meiosis, the transcription of *IME1, IME2*, and *NDT80* declined similarly to the wild-type strain (Figure 3), suggesting that Rpd3 possesses an additional function that is independent of Rco1 and Sds3, namely independent of the integrity of the Rpd3S and Rpd3L. This effect can be explained by two simple hypotheses as follows: 1) During late meiosis, Rpd3 is activated in a novel complex that does not include Sds3 or Rco1 and may include Dep1 that when absent led to increased transcription during late meiosis (Figure 3). We discounted this hypothesis, because mass spectrometry detected all of the integral components of Rpd3L and Rpd3S in cells cultured for 6 hours in SPM (Table S1). Interestingly, the only missing protein was Ume6, in agreement with a report that it is subject to Ime1-dependent degradation [65]. In contrast, RNA polymerase II, which recruits Rpd3S [20], is present. 2) Rpd3 possesses a novel transcriptional repressor activity that is independent of Sds3 and Rco1, but requires Dep1. Because the histone deacetylase activity of Rpd3 requires Rco1 and Sds3, the latter hypothesis predicts that cells expressing catalytically inactive Rpd3 will exhibit the same phenotype as the *sds3*Δ *rco1*Δ-double mutant. This hypothesis was validated, because the transcription of *IME1*, *IME2*, and *NDT80* during late meiosis declined in cells expressing the *rpd3H150AH151A* allele (Figure 3 and Table 4). During vegetative growth, repression of *IME1* and *IME2* was mediated by the catalytic activity of Rpd3L and the noncatalytic activity of Rpd3, whereas during late meiosis, the effect was mediated only by the latter. Histone H4 acetylation decreased during late meiosis (Figure 2), likely caused by the catalytic activity of Rpd3. This did not significantly affect transcription and possibly mediated by the counteracting effect of the noncatalytic activity of Rpd3. Our results support a recent *in vitro* observation demonstrating that Rpd3 possesses a catalytic function leading to deacetylated lysine residues and a noncatalytic activity that affects nucleosome stability [49]. Consistent with this scenario, mass spectrometry revealed that Rpd3 associated with proteins involved in chromatin remodeling (Table S1). Chen et al. reported that this activity requires only the core components of the Rpd3 complexes Rpd3, Sin3, and Ume1 [49]. Our results using the *rco1*Δ *sds3*Δ-double mutant agree, but the effect of deleting *DEP1* suggests that Dep1 is also required for the noncatalytic activity of Rpd3.

In summary, our analysis of meiosis-specific genes demonstrates that 1) the histone deacetylase activity of Rpd3 that is mediated by Rpd3S activates transcription, 2) in Rpd3L the catalytic activity of Rpd3 switches from negative to positive, depending on the availability of a nitrogen source, and 3) a noncatalytic activity of Rpd3 represses transcription.

## Supporting Information

Table S1 **Mass spectroscopic analysis of Rpd3 isolated from cells cultured in SPM for six hours.** Cells grown in SA to a density of 1 × 10^7^ cell/ml were shifted to SPM. After six hours in SPM, proteins were crosslinked with 1% formaldehyde for 15 min. Rpd3 complexes isolated by IP were subjected to gel electrophoresis, excised from the gel, and analyzed using mass spectroscopy. Strains: *MAT*a/*MAT*α *RPD3-13xmyc-URA3*/*RPD3-13xmyc-URA3* (Y1767) and *MAT*a/*MAT*α *rpd3*Δ*::HIS3*/*rpd3* Δ*::HIS3* (Y1888, control). The results for strain Y1767 are shown. Sf - score for each peptide was calculated by a neural network algorithm that incorporates the Xcorr, DeltaCn, Sp, RSp, peptide mass, charge state, and the number of matched peptides for the search. P (pep) displays the probability of finding a match as good as or better than a random match.
(DOCX)
