# Supplementary material for: The Roles of the Catalytic and Noncatalytic Activities of Rpd3L and Rpd3S in the Regulation of Gene Transcription in Yeast
Source: PLoS One. 2013 Dec 17;8(12):e85088. doi: 10.1371/journal.pone.0085088 (PMC3866184; doi:10.1371/journal.pone.0085088)
Supplement: Table S1 — Mass spectroscopic analysis of Rpd3 isolated from cells cultured in SPM for six hours. Cells grown in SA to a density of 1 × 10^7^ cell/ml were shifted to SPM. After six hours in SPM, proteins were crosslinked with 1% formaldehyde for 15 min. Rpd3 complexes isolated by IP were subjected to gel electrophoresis, excised from the gel, and analyzed using mass spectroscopy. Strains: MATa/MATα RPD3-13xmyc-URA3/RPD3-13xmyc-URA3 (Y1767) and MATa/MATα rpd3Δ::HIS3/rpd3 Δ::HIS3 (Y1888, control). The results for strain Y1767 are shown. Sf - score for each peptide was calculated by a neural network algorithm that incorporates the Xcorr, DeltaCn, Sp, RSp, peptide mass, charge state, and the number of matched peptides for the search. P (pep) displays the probability of finding a match as good as or better than a random match. (DOCX) [file pone.0085088.s001.docx]

**Table S1.** Mass spectrometric analysis of Rpd3 complexes in cells grown for 6 h in SPM

| **Protein** | **P (pep)** | **Sf** | **Function** |
| --- | --- | --- | --- |
| **HDAC complex** | | | |
| Sin3 | 4.37E-11 | 46.00 | Rpd3L/S |
| Rpd3 | 2.14E-12 | 22.21 | Rpd3L/S |
| Ume1 | 9.71E-08 | 7.86 | Rpd3L/S |
| Rco1 | 3.23E-09 | 14.02 | Rpd3S |
| Eaf3 | 6.90E-08 | 9.97 | Rpd3S |
| dep1 | 2.15E-07 | 7.56 | Rpd3L |
| Sds3 | 1.37E-06 | 6.97 | Rpd3L |
| Rxt2 | 3.16E-08 | 6.26 | Rpd3L |
| Rxt3 | 7.37E-13 | 6.26 | Rpd3L |
| Pho23 | 3.54E-06 | 6.03 | Rpd3L |
| Sap30 | 4.59E-06 | 2.43 | Rpd3L |
| Cti6 | 2.34E-11 | 1.86 | Rpd3L |
| **Chromatin Remodeling** | | | |
| Spt16 | 8.71E-11 | 8.95 | Subunit of the heterodimeric FACT complex |
| Cyc8 | 1.40E-07 | 5.27 | co-repressor and co-activator, recruits the SWI/SNF and SAGA complexes to promoters |
| Rsc8 | 2.33E-14 | 4.68 | RSC chromatin remodeling complex |
| Spt6 | 2.64E-08 | 4.08 | Nucleosome remodeling protein |
| Rvb2 | 2.34E-06 | 3.89 | chromatin remodeling complexes |
| Rvb1 | 5.57E-04 | 3.03 | chromatin remodeling |
| Isw1 | 9.12E-06 | 2.21 | chromatin remodelers |
| **Known and Possible Substrates** | | | |
| Hhf1 | 7.69E-07 | 4.51 | histone H4 |
| Htb2 | 2.24E-04 | 2.20 | Histone H2B |
| Tho1 | 6.45E-07 | 2.83 | nuclear RNA-binding protein; binds to transcribed chromatin |
| Rfc3 | 2.69E-07 | 2.66 | Replication factor C |
| Gal3 | 4.47E-05 | 2.35 | Transcriptional regulator; forms a complex with Gal80p to relieve Gal80p inhibition of Gal4p |
| **Transcriptional Elongation, substrate? Recruitment?** | | | |
| Rrp5 | 3.41E-08 | 20.52 | RNA binding protein with preference for single stranded tracts of U's involved in synthesis of both 18S and 5.8S r |
| Rpo21 | 8.65E-12 | 10.76 | RNA polymerase II largest subunit |
| Spt5 | 4.53E-10 | 7.73 | Roles in concert with RNA polymerases I and II, including regulation of transcription elongation |
| Rpa135 | 2.50E-11 | 5.23 | RNA polymerase I second largest subunit |
| Npl3 | 5.23E-08 | 4.22 | RNA-binding protein; promotes elongation |
| Rpb2 | 1.04E-08 | 2.95 | RNA polymerase II second largest subunit |
| Tho2 | 8.87E-06 | 2.54 | THO complex, which is required for efficient transcription elongation |
| Dst1 | 7.62E-06 | 2.18 | General transcription elongation factor TFIIS |
| Rrp5 | 3.41E-08 | 20.52 | RNA binding protein with preference for single stranded tracts of U's involved in synthesis of both 18S and 5.8S r |
| **mRNA Processing** | | | |
| Sub2 | 2.05E-08 | 8.48 | Component of the TREX complex required for nuclear mRNA export |
| Dbp2 | 1.46E-08 | 8.59 | RNA helicase of the DEAD-box protein family, involved in nonsense-mediated mRNA decay and rRNA processing |
| Dbp3 | 4.10E-10 | 5.06 | RNA-Dependent ATPase, member of DExD/H-box family |
| Dbp10 | 3.66E-07 | 5.75 | Putative ATP-dependent RNA helicase of the DEAD-box protein family |
| Rse1 | 3.63E-05 | 5.26 | involved in pre-mRNA splicing |
| Gbp2 | 1.10E-07 | 3.39 | Poly(A+) RNA-binding protein, involved in the export of mRNAs |
| Sto1 | 1.32E-08 | 3.80 | Large subunit of the nuclear mRNA cap-binding protein complex |
| Cft1 | 8.15E-08 | 2.37 | RNA-binding subunit of the mRNA cleavage and polyadenylation facto |
| Prp43 | 5.93E-04 | 2.35 | RNA helicase in the DEAH-box family, functions in both RNA polymerase I and polymerase II transcript metabolism |
| **rRNA Processing** | | | |
| Utp10 | 2.85E-07 | 9.01 | Nucleolar protein, involved in processing of pre-18S rRNA |
| Utp22 | 1.21E-08 | 8.14 | maturation of pre-18S rRNA |
| Utp21 | 1.01E-04 | 2.86 | involved in production of 18S rRNA and assembly of small ribosomal subunit |
| Nop2 | 6.63E-06 | 7.85 | processing and maturation of 27S pre-rRNA |
| Nop12 | 1.26E-04 | 2.94 | involved in pre-25S rRNA processing |
| Nop14 | 2.66E-05 | 3.62 | maturation and nuclear export of 40S ribosomal subunits |
| Dbp9 | 3.51E-05 | 6.45 | DEAD-box protein required for 27S rRNA processing |
| Nog1 | 1.33E-05 | 5.78 | required for 60S ribosomal subunit biogenesis |
| Rpl3 | 5.65E-04 | 4.76 | Ribosomal 60S subunit protein |
| Nug1 | 6.11E-09 | 3.68 | GTPase that associates with nuclear 60S pre-ribosomes |
| Rpl8A | 1.08E-06 | 2.69 | Ribosomal 60S subunit protein L8A; |
| Rpl4b | 6.07E-08 | 2.58 | Ribosomal 60S subunit protein |
| Urb1 | 3.00E-05 | 5.14 | Nucleolar protein required for the normal accumulation of 25S and 5.8S rRNAs, |
| Ssb1 | 3.24E-11 | 4.90 | ribosome-associated molecular chaperone |
| Erb1 | 3.60E-08 | 4.42 | Constituent of 66S pre-ribosomal particles |
| Ola1 | 1.69E-05 | 4.37 | maturation of the large ribosomal subunit; |
| Pwp2 | 8.84E-07 | 4.41 | pre-ribosomal component |
| Nsr1 | 5.22E-06 | 3.58 | required for pre-rRNA processing |
| Drs1 | 2.39E-06 | 3.23 | DEAD-box protein required for ribosome assembly and function |
| Kre33 | 1.00E-04 | 3.23 | biogenesis of the small ribosomal subunit |
| Nan1 | 3.62E-04 | 2.41 | component of the small (ribosomal) subunit |
| Noc2 | 6.56E-07 | 2.58 | mediate intranuclear transport of ribosomal precursors |
| Urb2 | 2.57E-05 | 1.87 | required for normal metabolism of the rRNA primary transcript |
| **Translation** | | | |
| Tef2 | 4.49E-06 | 7.11 | translation elongation factor |
| Eft2 | 3.96E-09 | 7.90 | Elongation factor |
| Rpg1 | 3.99E-11 | 6.12 | eIF3a subunit of the core complex of translation initiation factor |
| Prt1 | 3.26E-07 | 5.54 | eIF3b subunit of the core complex of translation initiation factor 3 |
| Tif2 | 2.11E-06 | 3.44 | Translation initiation factor eIF4A |
| Ded1 | 2.48E-06 | 3.33 | RNA helicase, required for translation initiation of all yeast mRNAs |
| Yef3 | 2.52E-06 | 2.73 | Gamma subunit of translational elongation factor eEF1B |
| Hyp2 | 1.33E-04 | 1.78 | Translation elongation factor eIF-5A |
| **various functions** | | | |
| Hsc82 | 8.94E-09 | 18.36 | Cytoplasmic chaperone of the Hsp90 family |
| Ssa2 | 1.97E-11 | 15.22 | member of heat shock protein 70 |
| Hsp104 | 3.55E-08 | 10.34 | heat shock |
| Cdc48 | 7.21E-05 | 6.62 | AAA ATPase involved in multiple processes |
| Pma2 | 3.48E-09 | 6.03 | Plasma membrane H+-ATPase |
| Fun19 | 6.67E-09 | 6.14 | unknown function |
| Rbp2 | 5.52E-08 | 5.78 | Cytoplasmic RNA-binding protein |
| Cct8 | 8.64E-08 | 5.65 | cytosolic chaperonin |
| Cct3 | 3.90E-05 | 5.29 | cytosolic chaperonin |
| Cct2 | 1.45E-06 | 2.77 | cytosolic chaperonin |
| Cct4 | 1.16E-06 | 1.70 | cytosolic chaperonin |
| Cct7 | 8.57E-06 | 1.49 | cytosolic chaperonin |
| Ssa1 | 2.84E-08 | 4.94 | ATPase involved in protein folding and NLS-directed nuclear transport |
| Act1 | 6.36E-08 | 4.08 | actin |
| Pet9 | 2.19E-06 | 3.78 | mitochondrial inner membrane |
| Ald4 | 2.41E-05 | 3.19 | Mitochondrial aldehyde dehydrogenase |
| ATP1 | 1.03E-08 | 2.74 | Alpha subunit of the F1 sector of mitochondrial F1F0 ATP synthase |
| Bmh2 | 1.82E-07 | 3.28 | 14-3-3 protein |
| Bre1 | 3.74E-04 | 2.36 | E3 ubiquitin ligase |
| Ubi4 | 6.50E-05 | 2.11 | Ubiquitin |
| **various enzymes** | | | |
| Gal1 | 3.21E-09 | 17.00 | Galactokinas |
| Lcl1 | 3.41E-08 | 11.55 | Isocitrate lyase |
| Ura1 | 8.32E-09 | 10.29 | Glutamine-dependent carbamoyl-phosphate synthase |
| Lys20 | 1.44E-08 | 10.05 | Homocitrate synthase isozyme |
| Aco1 | 6.34E-09 | 8.19 | Aconitase |
| Acc1 | 4.06E-10 | 7.46 | Acetyl-CoA carboxylase |
| Pck1 | 1.68E-06 | 7.28 | Phosphoenolpyruvate carboxykinas |
| Acs2 | 2.24E-09 | 6.04 | Acetyl-coA synthetase isoform |
| Gal10 | 3.23E-06 | 6.70 | UDP-glucose-4-epimerase |
| Cdc19 | 3.56E-04 | 6.28 | Pyruvate kinase |
| Erg13 | 7.52E-09 | 5.16 | HMG-CoA synthase |
| Pgk1 | 7.89E-07 | 5.50 | 3-phosphoglycerate kinase |
| Tal1 | 1.91E-08 | 6.02 | Transaldolase |
| Adh2 | 8.82E-10 | 4.54 | Glucose-repressible alcohol dehydrogenase II |
| Fox2 | 1.40E-05 | 5.38 | enzyme of the peroxisomal fatty acid beta-oxidation pathway |
| Adh1 | 2.18E-10 | 5.33 | Alcohol dehydrogenase |
| Tkl1 | 8.19E-05 | 3.61 | Transketolase |
| Fpr3 | 3.63E-09 | 3.27 | Nucleolar peptidyl-prolyl cis-trans isomerase |
| Mdh2 | 1.29E-07 | 3.39 | malate dehydrogenase |
| Gln1 | 6.77E-09 | 3.05 | Glutamine synthetase |
| Gnd1 | 6.39E-09 | 3.16 | 6-phosphogluconate dehydrogenase |
| Sah1 | 4.34E-06 | 3.27 | S-adenosyl-L-homocysteine hydrolase |
| Ade3 | 1.10E-08 | 3.37 | trifunctional enzyme C1-tetrahydrofolate synthase |
| Fba1 | 2.42E-09 | 2.78 | Fructose 1,6-bisphosphate aldolase |
| Ino1 | 7.12E-08 | 2.84 | Inositol-3-phosphate synthase |
| Cpr1 | 4.36E-07 | 2.73 | Cytoplasmic peptidyl-prolyl cis-trans isomerase |
| Ncl1 | 4.06E-06 | 2.41 | S-adenosyl-L-methionine-dependent tRNA: m5C-methyltransferase |
| Leu1 | 3.91E-05 | 2.20 | Isopropylmalate isomerase |
| Tsa1 | 1.17E-05 | 2.19 | Thioredoxin peroxidase |
| Tdh2 | 1.53E-06 | 2.17 | Glyceraldehyde-3-phosphate dehydrogenase |
| Asn1 | 7.01E-05 | 2.45 | Asparagine synthetase |
| Lys21 | 4.44E-04 | 2.22 | Homocitrate synthase isozyme |

Cells grown in SA to 1 × 10^7^ cell/ml were shifted to SPM. After 6 h in SPM, proteins were cross-linked by treatment with 1% formaldehyde for 15 min. Rpd3 complexes isolated by IP were electrophoresed, excised from the gels, and analyzed using MS. Strains: *MAT*a/*MAT*α *RPD3-13xmyc-URA3*/*RPD3-13xmyc-URA3* (Y1767) *MAT*a/*MAT*α and *rpd3Δ::HIS3*/*rpd3::HIS3* (Y1888, control). Results represent proteins detected in strain Y1767. Sf, score for each peptide was calculated by a neural network algorithm that incorporates the Xcorr, DeltaCn, Sp, RSp, peptide mass, charge state, and the number of matched peptides for the search. P (pep) displays the probability of finding a match as good as or better than a random match.
